# Isolation and Characterization of Chinese Standard Fulvic Acid Sub-fractions Separated from Forest Soil by Stepwise Elution with Pyrophosphate Buffer

**DOI:** 10.1038/srep08723

**Published:** 2015-03-04

**Authors:** Yingchen Bai, Fengchang Wu, Baoshan Xing, Wei Meng, Guolan Shi, Yan Ma, John P. Giesy

**Affiliations:** 1 State Key Laboratory of Environmental Criteria and Risk Assessment, Chinese Research Academy of Environmental Sciences, Beijing, 100012, China; 2 Stockbridge School of Agriculture, University of Massachusetts, Amherst, MA, 01003, USA; 3 Lanzhou New Area Environmental Protection Bureau, Lanzhou, 730314, China; 4 Research Center of Environmental Biology and Green Chemistry, School of Environmental and Municipal Engineering, Qingdao Technological University, Qingdao, 266033, China; 5 Department of Biomedical and Veterinary Biosciences and Toxicology Centre, University of Saskatchewan, Saskatoon, Saskatchewan, Canada; 6 Department of Biology and Chemistry, and State Key Laboratory for Marine Pollution, City University of Hong Kong, Kowloon, Hong Kong, China; 7 State Key Laboratory of Pollution Control and Resource Reuse, School of the Environment, Nanjing University, Nanjing, 210046, China; 8 Zoology Department, National Food Safety and Toxicology Center, and Center for Integrative Toxicology, Michigan State University, East Lansing, 48824, USA

## Abstract

XAD-8 adsorption technique coupled with stepwise elution using pyrophosphate buffers with initial pH values of 3, 5, 7, 9, and 13 was developed to isolate Chinese standard fulvic acid (FA) and then separated the FA into five sub-fractions: FA_pH3_, FA_pH5_, FA_pH7_, FA_pH9_ and FA_pH13_, respectively. Mass percentages of FA_pH3_-FA_pH13_ decreased from 42% to 2.5%, and the recovery ratios ranged from 99.0% to 99.5%. Earlier eluting sub-fractions contained greater proportions of carboxylic groups with greater polarity and molecular mass, and later eluting sub-fractions had greater phenolic and aliphatic content. Protein-like components, as well as amorphous and crystalline poly(methylene)-containing components were enriched using neutral and basic buffers. Three main mechanisms likely affect stepwise elution of humic components from XAD-8 resin with pyrophosphate buffers including: 1) the carboxylic-rich sub-fractions are deprotonated at lower pH values and eluted earlier, while phenolic-rich sub-fractions are deprotonated at greater pH values and eluted later. 2) protein or protein-like components can be desorbed and eluted by use of stepwise elution as progressively greater pH values exceed their isoelectric points. 3) size exclusion affects elution of FA sub-fractions. Successful isolation of FA sub-fractions will benefit exploration of the origin, structure, evolution and the investigation of interactions with environmental contaminants.

Humic substances are complex, heterogeneous, mixtures of organic compounds with different functional groups, and their molecular masses (MM) are still operationally defined and being debated^1^,^2^,^3^,^4^. Humic substances can be operationally defined into fulvic acid (FA; soluble at all pH values), humic acid (HA; soluble in alkaline media and insoluble at pH 1.0), and humin (insoluble at all pH values) according to their water solubility^2^,^3^,^4^,^5^. Among these three groups, FAs have the least molecular masses and are the most mobile fraction of humic substances^1^. Some researchers have reported that FAs have potential effects on the bioavailability and transport of nutrients^5^, heavy metals^6^,^7^,^8^,^9^,^10^,^11^,^12^,^13^, polycyclic aromatic hydrocarbons^14^,^15^ and other chemicals^16^. Despite efforts for more than 200 years, that have applied a wide range of techniques to characterize FAs, their structures and functions are still not well understood. Proper separation and characterization of FAs is critical to further elucidate their structures and mechanisms of interactions with environmental contaminants. Separation of FA into fractions with different chemical properties to reduce its heterogeneity remains challenging.

At present, various separation techniques including resin techniques (e.g., XAD-series resins and diethylaminoethyl cellulose), chromatographic techniques (such as reversed-phase liquid chromatography and high performance size exclusion chromatography), ultra-filtration, and capillary electrophoresis have been used to separate and characterize FAs^2^,^3^. Of those methods, the International Humic Substances Society (IHSS) suggests using the XAD-8 resin adsorption technique as a standard method for isolation and purification of FAs from solid phase source materials^2^. This resin has a large adsorption capacity, and the adsorbed humic substances are partly recovered by eluting them with basic solutions. By updating the XAD-8 adsorption technique, a stepwise elution has also been employed to separate soil HAs and FAs after adsorption on XAD-8 resin. For example, soil HAs were successfully fractionated into four sub-fractions using a stepwise elution with universal buffers with initial pH 7 and 11, water, and 50% ethanol as early as 1990^17^. Four sub-fractions were separated based on the number of rings of aromatic groups, length of aliphatic substituents, and the content of carboxylic and phenol groups in HA molecules^17^. Another stepwise elution of soil was used to separated FAs into five sub-fractions with universal buffers at pH 4.8, 7 and 11, water, and ethanol^5^. It suggested that the content of carboxylic and aliphatic C regulated elution of FA sub-fractions^5^. Recently, successful fractionation of soil FAs was accomplished by use of stepwise elution from XAD-8 with 0.01 mol/L HCl, 0.01 mol/L HCl + 20% methanol, 0.01 M HCl + 40% methanol, and 100% methanol. The decreasing polarity was reported with elution sequence^18^. Although they have been incrementally improved, early methods for stepwise elution applied universal buffers and organic compounds including acetic acid, methanol, and ethanol^5^,^17^. However, it is difficult to quantitatively evaluate effects of these various solvents on compositions of fractions and possible effects on structure of FA during stepwise elution^19^. Pyrophosphate buffers that can maintain a range of pH were considered suitable to adjust extraction solutions to pH 3, 5, 7, 9, and 13^20^. Possible residues of pyrophosphate in extracted and fractionated FA can easily be evaluated by measuring the content of phosphorus. Therefore, pyrophosphate buffers were applied instead of universal buffers and organic solvents to fractionate FA.

Even though XAD-8 resin contains slightly polar ester groups and carries a slight negative charge on its surfaces, it is basically hydrophobic in nature^21^,^22^,^23^. Humic substances become sufficiently hydrophobic to be adsorbed on the surface of XAD-8 resin at pH 1.0 or 2.0, which are the pH values recommended by the IHSS to adsorb FAs on XAD-8 resin^21^,^22^,^23^. Humic mixture become sufficiently hydrophilic and carry negative charges to be desorbed from surfaces of XAD-8 resin at pH 13^21^,^22^,^23^. Therefore, a pH of 13 is recommended by IHSS to elute adsorbed FA from XAD-8 resin. However, possible mechanisms of stepwise elution for FA adsorbed XAD-8 resin were still unknown. In order to improve the separation methods, these mechanisms need to be investigated.

The FA isolated from soil of Jiufeng forest was recommended as the Chinese standard fulvic acid (CSFA)^19^. The objectives of the current work with CSFA were to: 1) develop an improved method for isolation and stepwise elution after FA was adsorbed on XAD-8 resin; 2) fractionate FA and characterize the chemical structure of its sub-fractions; and 3) elucidate possible mechanisms of stepwise elution.

## Results

### Fractionation of CSFA

**Figure 1:**
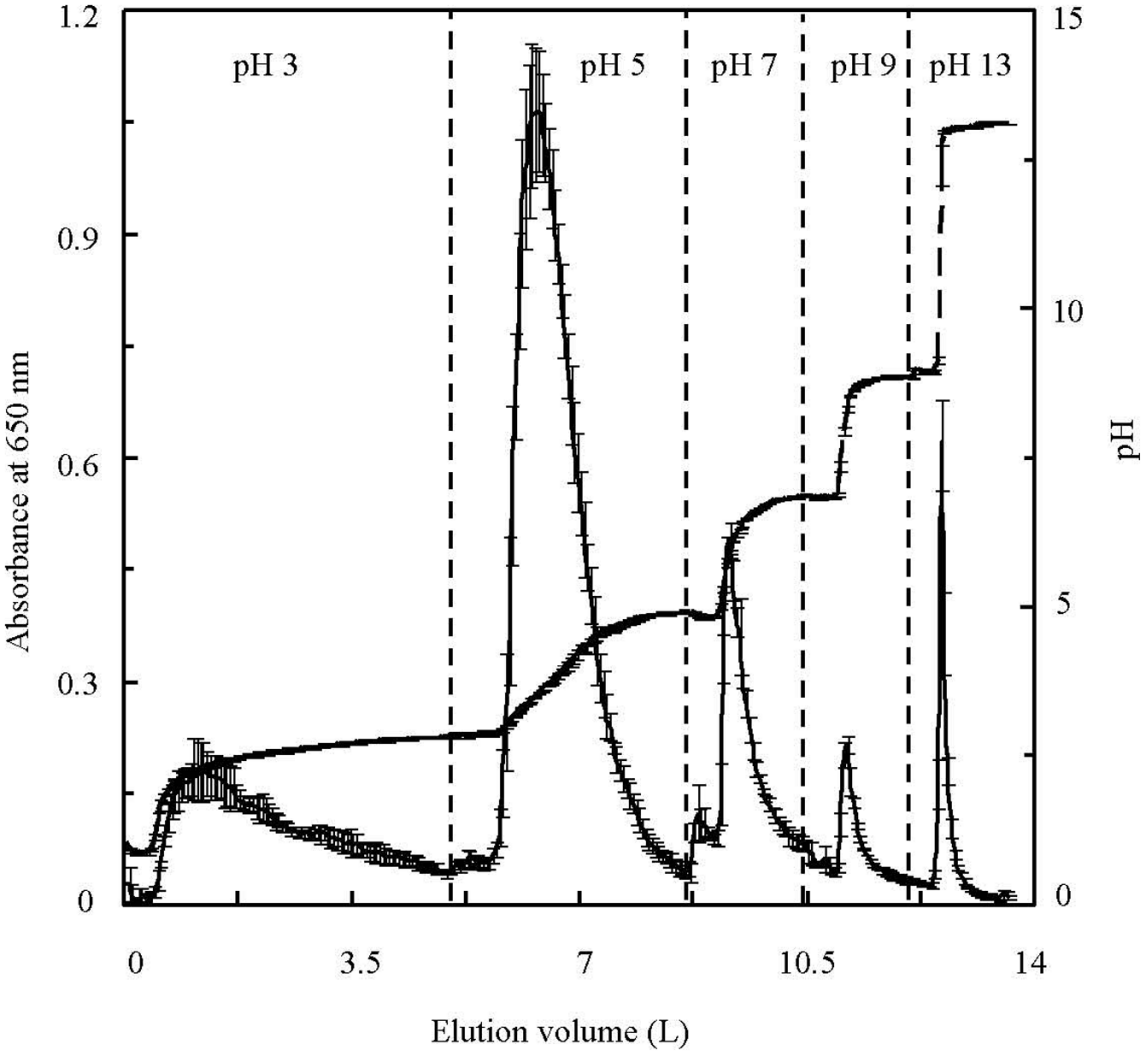
The curves of XAD-8 stepwise elution using pyrophosphate buffers with initial pH 3, 5, 7, 9, and 13. The solid line indicates absorbance at 650 nm of eluate, and the dashed line represents pH change.

Stepwise elution of CSFA using pyrophosphate buffers with initial pH of 3, 5, 7, 9, and 13 was performed, and CSFA sub-fractions were named: FA_pH3_, FA_pH5_, FA_pH7_, FA_pH9_, and FA_pH13_ respectively. A significant relationship exists between the UV-Vis absorbance at 650 nm and the concentration of CSFA or its sub-fractions with dilution (p<0.05). During stepwise elution of CSFA from XAD-8, there was one maximum peak detected with an absorbance at 650 nm for each of the buffer with initial pH of 3, 5, 7, 9 and 13 (Fig. 1). Maximal absorbance peaks ranged from 1.06 to 0.18. Widths of peaks decreased from 5.0 to 1.5 L with elution sequence from FA_pH3_ to FA_pH13_ (Supplementary Table S1). The mass proportion of CSFA sub-fractions decreased from 42.2% to 2.5% with elution sequence (Supplementary Table S1). Earlier eluting sub-fractions (EESF), including FA_pH3_ and FA_pH5_ accounted for approximately 80% of the total mass of CSFA after freeze-drying. Later-eluting sub-fractions (LESF), including FA_pH9_ and FA_pH13_ accounted for less than 8%. The five sub-fractions accounted for 99.0–99.5% of the total mass of CSFA.

### Elemental compositions of CSFA and its sub-fractions

Mass percentages and atomic ratios of CSFA sub-fractions are summarized in Table 1. Proportions of C and H increased from 44.23% to 53.04% and from 3.81% to 6.19%, respectively; while O content decreased from 47.79% to 36.04% as a function of the elution sequence (Table 1). Mean content of N in LESF (2.96–3.11%) expressed on a mass basis, was 38.1% greater than those in EESF (4.18–4.20%). The content of N in FA_pH7_ (3.29%) was larger than that in LESF but lesser than that in EESF. Total P content, expressed as pyrophosphate, in CSFA and its sub-fractions was less than 0.3% (Table 1). Ash content in CSFA and its sub-fractions ranged from 0.23 to 0.49%. H/C ratios of CSFA sub-fractions increased from 1.03 to 1.39, while O/C ratios decreased from 0.81 to 0.50 with the elution sequence. The polarity ratio of FA can be evaluated using the atomic ratio of (O+N)/C while ignoring the small amount of S, P, and ash that were generally less than 1% by mass. The polarity ratios decreased from 87.1% to 57.4% with the elution sequence (Table 1).

**Figure 2:**
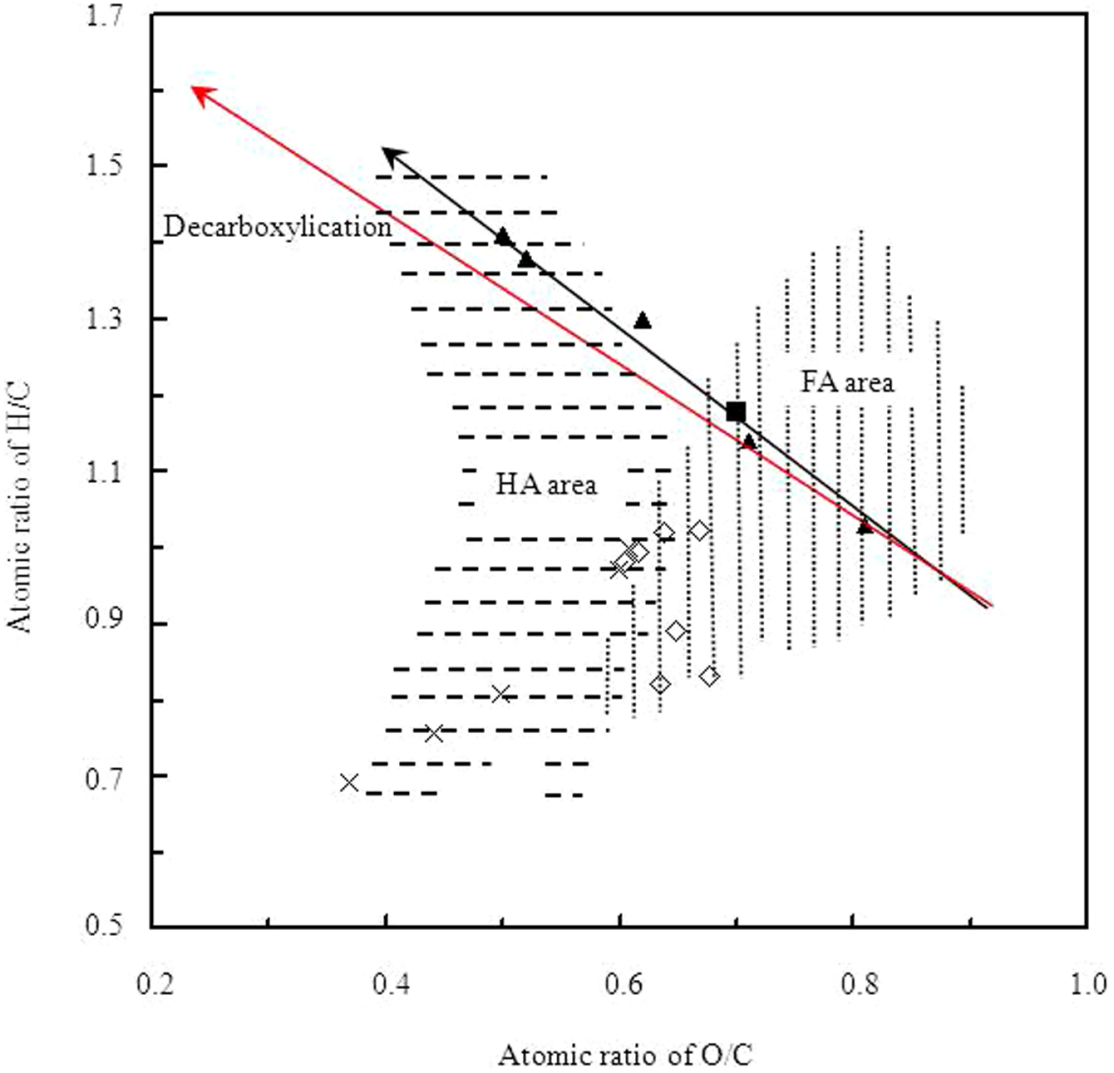
Position of fulvic and humic acids in a van Krevelen diagram. 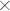
 Standard humic acids from IHSS, 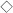 standard FAs from IHSS, 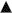 CSFA sub-fractions, 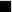 CSFA. Standard HAs from IHSS include Suwannee River I standard HA, Suwannee River II standard HA, Elliott Soil standard HA, Pahokee Peat standard HA, and Leonardite standard HA. Standard FAs from IHSS include Suwannee River I standard FA, Suwannee River II standard FA, Elliott Soil I standard FA, Elliott Soil II standard FA, Elliott Soil III standard FA, Pahokee Peat I standard FA, and Pahokee Peat II standard FA.

The van Krevelen diagram was developed to illustrate the coalification process, and recently it was successfully used to compare elemental compositions of coals, humic substances, and plant constituents^17^,^24^,^25^,^26^. The van Krevelen plots for CSFA and its sub-fractions, as well as standard FAs and HAs recommended by IHSS are presented in Fig. 2. The standard FAs and HAs recommended by IHSS were located in the FA and HA areas of the van Krevelen diagram, respectively (Fig. 2). EESF were located in the FA area, however LESF were located in the HA area. FA_pH7_ was located in the transitional area between FA and HA areas of the van Krevelen diagram. A line with a slope of about -1 was observed from FA_pH3_ to FA_pH13_ in van Krevelen diagram (black arrow in Fig. 2). The decarboxylication is displayed with a slope of -1 in van Krevelen diagram (red arrow in Fig. 2).

### FTIR spectra of CSFA and its sub-fractions

**Figure 3:**
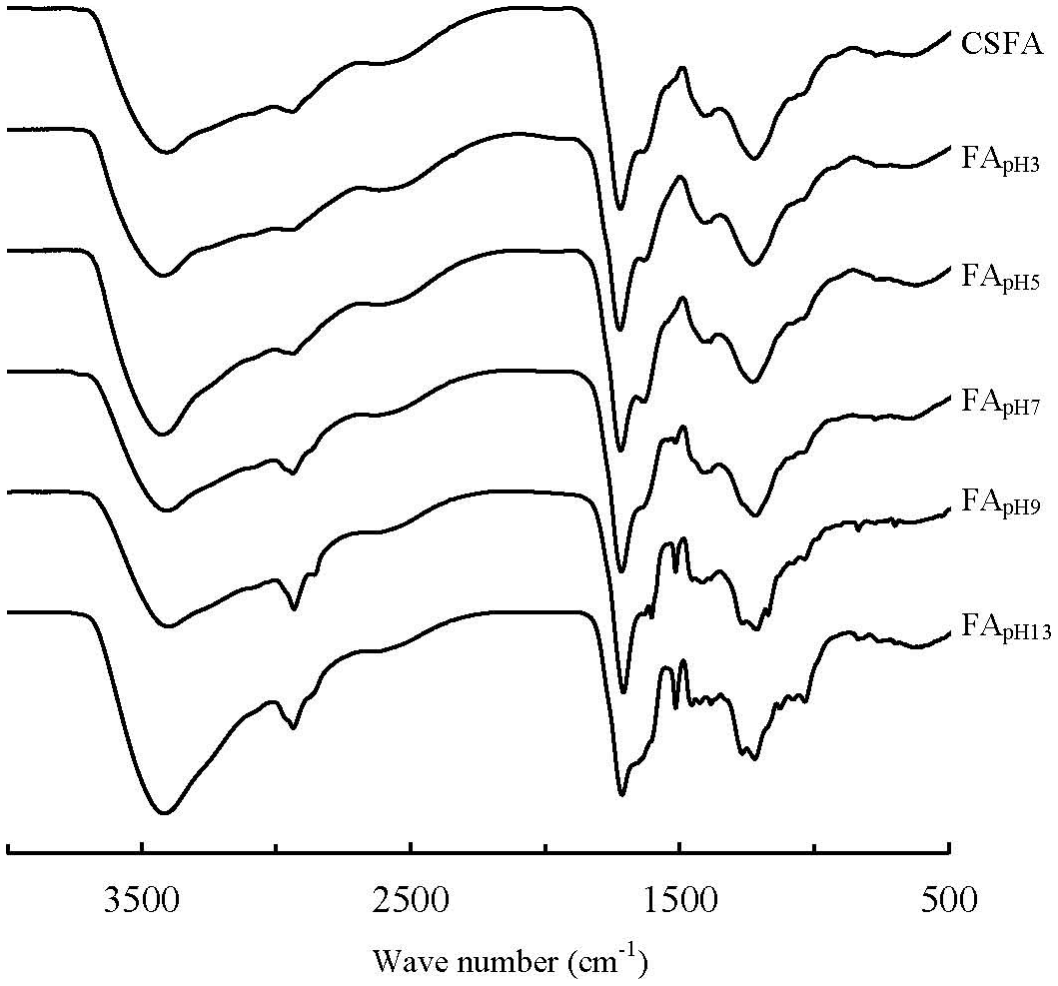
FTIR spectra of CSFA and its sub-fractions.

Both CSFA and its sub-fractions exhibited four strong bands in FTIR spectra, which were associated with O-H stretching (3500–3300 cm^−1^), aliphatic C-H stretching (2920 cm^−1^), C = O stretching of carboxylic/carbonyl groups (1720 cm^−1^), and C-O stretching of carboxylic groups, phenols, and unsaturated ethers (1230 cm^−1^) (Fig. 3)^27^. An amide band I (C = O stretching of amide groups) at about 1640 cm^−1^ and amide band II (N-H deformation and C = N stretching) at approximately 1520 cm^−1^ were observed in FA_pH7_ and LESF (Fig. 3). Peaks located at 1420 cm^−1^ were observed for EESF, FA_pH7_ and CSFA that can be attributed to the antisymmetric stretching of -COOH groups. However, for LESF, in the region of 1460–1360 cm^−1^, multi-peaks appeared depicting the C-H bending vibration of methylene, methyl, isopropyl, or tertiary butyl groups^5^,^27^,^28^.

Although absorption or transmittance magnitudes of FTIR spectra cannot be compared directly, peak height ratios of FTIR spectra have been used to interpret the relative structural change of humic substances^5^,^28^. The ratios of the absorbance at 2920 cm^−1^ and 1720 cm^−1^ (A_2920_/A_1720_) were related to changing of aliphatic C-H groups to C = O groups in FAs. The A_2920_/A_1720_ increased from 0.51 to 0.63 with elution sequence (Table 1).

### ^13^C-NMR spectra of CSFA and its sub-fractions

**Figure 4:**
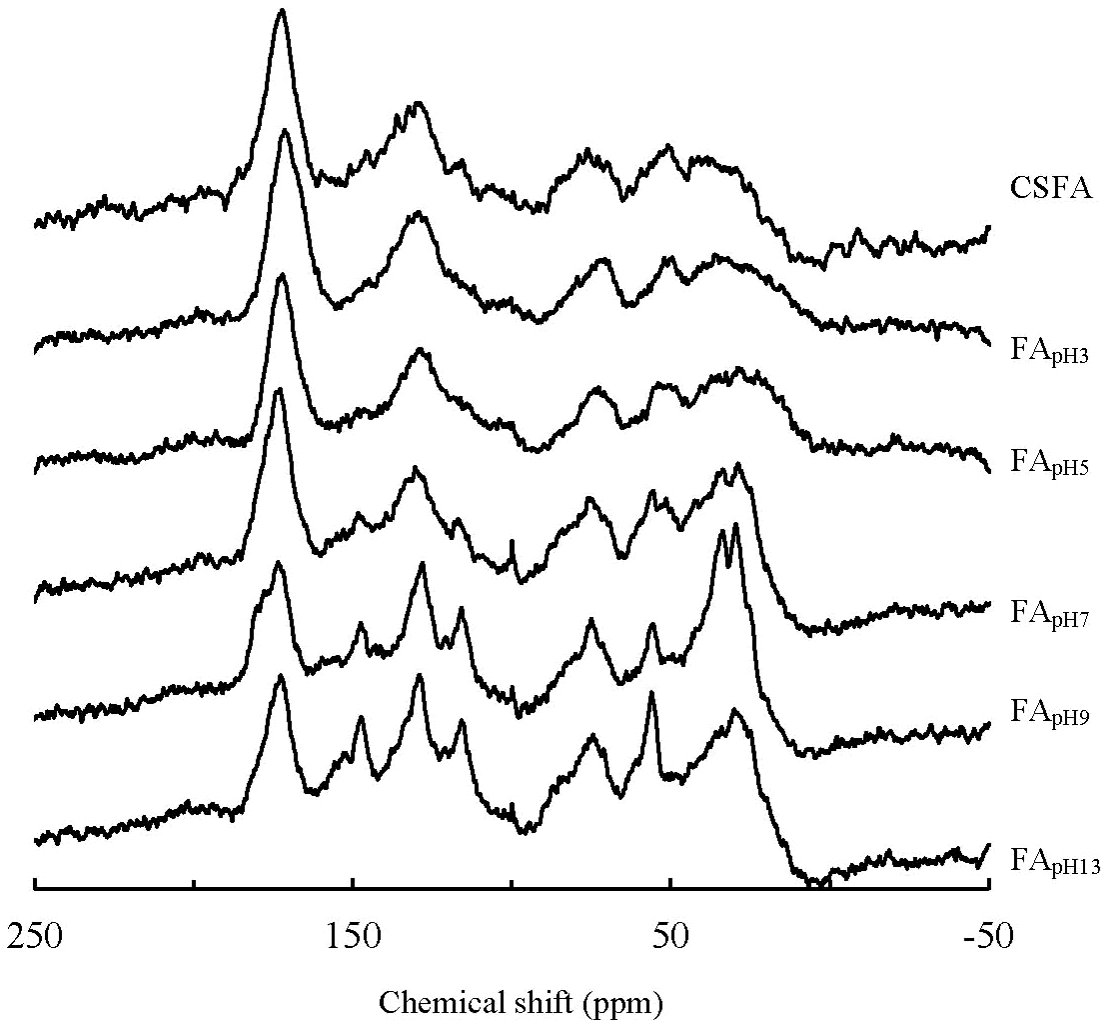
^13^C-NMR spectra of CSFA and its sub-fractions.

Typical peaks in the ^13^C-NMR spectra of CSFA and its sub-fractions exhibited the following shifts: alkyl C (30–35 ppm), methoxyl C (53 ppm), O-alkyl C (72 ppm), acetal C (100 ppm), aromatic C (117 and 130 ppm), phenolic C (147 ppm), carboxylic C (172 ppm), and carbonyl C (190–220 ppm) (Fig. 4)^5^,^18^,^28^. Peaks at 30–35 ppm are associated with alkyl C components including methyl, methylene and methyl^18^. EESF showed a weak, broad signal around 30–35 ppm, however FA_pH7_ and LESF had strong double-peaks at 30 ppm and 34 ppm (Fig. 4). The most prominent difference among the fractions occurred in the region between 110 and 160 ppm that is assigned to the aromatic carbons. Peaks around 117 and 130 are assigned to protonated and unprotonated aromatic C, respectively. Peaks at 117 and 147 ppm existed as two shoulders of peak at 130 ppm for FA_pH3_, and they became stronger from FA_pH3_ to FA_pH13_ (Fig. 4). The strong, sharp peaks near 172 ppm successively weakened from FA_pH3_ to FA_pH13_.

The alkyl C, methoxyl C, O-alkyl C, acetal C, aromatic C, phenolic C, carboxylic C, and carbonyl C ranged 17.7–32.2%, 8.2–11.5%, 10.2–11.7%, 3.5–5.2%, 19.7–22.7%, 5.8–8.2%, 13.0–23.3%, and 2.6–3.7% respectively, for CSFA sub-fractions estimated from ^13^C-NMR spectra. Carboxylic C decreased from 23.3% to 13.0% with elution sequence (Table 2). Mean carboxylic C of EESF was 62.7% larger than that of LESF (Table 2). The phenolic C increased from FA_pH3_ to FA_pH9_, and slightly decreased for FA_pH13_. Mean proportion of phenolic-C of LESF was 30.8% larger than that of EESF. The O-containing groups including methoxyl, O-alkyl, acetal and carbonyl C increased inversely with phenolic component from FA_pH9_ to FA_pH13_. The proportion of alkyl-C increased from 17.7% to 27.0% with elution sequence. Aliphatic-C ratios (including alkyl, methoxyl, O-alkyl, and acetal C) increased from 44.6% to 54.3% with elution sequence, which was associated with components of lesser polarity (Table 2).

### Molecular mass of CSFA and its sub-fractions

The number-averaged MM and mass-averaged MM were calculated according to the methods derived by Chin et al.^29^ and Yue et al.^30^. MM decreased from 3814 to 1745 Dalton (number-averaged MM) and from 4432 to 3369 Dalton (mass-averaged MM) with elution sequence (Table 3). The molecular mass dispersion increased from 1.16 to 1.92 with eluting sequence. It has been indicated that there were significant correlations between UV-vis absorbance at 280 nm, aromatic content, and MM^29^. However, no significant correlation was observed between these parameters in the present study.

### Fluorescence spectra of CSFA and its sub-fractions

Three dimensional excitation-emission fluorescence spectra are widely used to characterize humic substances. The UV-vis spectra of CSFA and its sub-fractions are shown in Supplementary Fig. S1. UV-vis absorbance decreased exponentially with increasing wavelength for CSFA and its sub-fractions (Fig. S1). However, a shoulder at around 270 nm appeared and increased from FA_pH7_ to LEFA (Fig. S1). Correction with UV-vis data, two major fluorescence peaks occurred for CSFA and EESF identified as peaks A and B (Table 3, Supplementary Fig. S2). In fluorescence spectra, peaks A and B are associated with conjugated unsaturated bond systems bearing carbonyl and carboxylic groups, respectively^2^,^32^,^33^. For FA_pH7_, four peaks were observed including two peaks in addition to peaks A and B, peaks C and D (Table 3, Supplementary Fig. S2). Peak B was observed in FA_pH7_, but not in LESF. Peak C is related to soluble microbial byproduct-like component, while peak D is related to simple aromatic proteins such as tyrosine (Supplementary Fig. S2)^32^,^33^. The excitation wavelength of peaks C and D were at 215–225 nm and 270–275 nm, respectively, while their emission wavelength was in the range of 300 to 340 nm.

Commonly, only certain fluorescence intensities and corresponding peak locations are used to investigate fluorescence spectra that contain more than 1,000 data points. In addition, some contour plots for peaks, such as peaks A and C, were obscured by Raman and/or Rayleigh scattering, which might affect fluorescence intensity and location of fluorescence peaks. The fluorescence regional integration method can quantitatively evaluate the ratios of different fluorescence-related components that emit fluorescence in different regions^32^,^33^. Peaks A, B, C, and D fell into four regions named as the A, B, C, and D region, respectively. The percentage fluorescence response (*P*_i_, i is the peak name of the region) was calculated using the method derived by Chen et al.^32^. The *P*_A_, *P*_B_, *P*_C_, and *P*_D_ ranged 15.0–19.9%, 51.1–71.9%, 4.5–12.1%, and 4.7–21.8% for CSFA sub-fractions from FA_pH3_ to FA_pH13_, respectively. The greatest percentage of *P*_B_ occurred for CSFA and its sub-fractions (Supplementary Fig. S3). The *P*_B_ decreased from 71.9% to 52.1% with elution sequence. The average *P*_A_ of EESF and FA_pH7_ was 23% larger than that of LESF (Supplementary Fig. S3). The average *P*_C_ of LESF was 2-fold larger than that of EESF and FA_pH7_, while mean *P*_D_ of LESF was 3-fold larger than that of EESF and FA_pH7_ (Supplementary Fig. S3).

## Discussion

### Physical-chemical properties of CSFA sub-fractions with elution sequence

The increasing H/C ratios and aliphatic-C ratio, as well as the decreasing O/C ratios and polarity ratios of CSFA sub-fractions showed the decrease of polarity and enrichment of the saturated aliphatic moieties instead of O-containing ones with elution sequence (Tables 1–2). The different atomic ratios are due to differences in functional groups and can be used to indirectly elucidate structural properties. For example, H/C ratios between 0.7 and 1.5 correspond to component of which the basic unit consists of an aromatic nucleus with an aliphatic side chain^17^,^24^,^25^,^26^,^34^. The H/C ratios of CSFA sub-fractions ranging from 1.30 to 1.39 were indicative of each CSFA sub-fraction containing both aromatic and aliphatic moieties (Table 1). In addition, large ratios of H/C of LESF implied enrichment of saturated aliphatic moieties with greater H saturation^5^. Alternatively, the smaller ratios of O/C of LESF indicated the presence of fewer O-containing functional groups^28^. The decreases of O/C ratios with the increasing pH were also observed during stepwise extractions of paddy soil FAs^5^. In ^13^C-NMR spectra, the intensity increase of peaks at 30–35 ppm also indicates greater proportions of saturated alkyl C in LESF than EESF (Fig. 4).

In van Krevelen diagram, HAs normally occupy a broad J-shaped area in the diagram with H/C ratios ranging from 0.5 to 1.5 and O/C atomic ratios of between 0.35 and 0.55 (transverse dashed line area in Fig. 2)^17^,^24^,^25^,^26^. FAs occupy a wide area to the right of HAs, which are indicated to be oxygen-rich compounds (vertical dashed lines area in Fig. 2)^17^,^24^,^25^,^26^. The illogical location for LESF in HA area indicated that LESF had a composition and/or structure similar to HA. The exponential decrease of UV-vis absorbance with the increase of wavelength was also reported for standard FAs recommended by IHSS^31^. Appearance of the shoulder peaks at 270 nm likely demonstrated the characteristics of the HAs in the FA_pH7_ and LESF. At least 20% of the recovery, including FA_pH7_ and LEFA contained both FA-like and HA-like components.

FAs contain two main acidic groups (carboxylic and phenol groups)^2^. Carboxylic group-containing component decreased as a function of elution sequence according to Van Krevelen diagram, FTIR spectra, and ^13^C-NMR spectra. The phenolic group-containing component increased and then decreased with elution sequence, and showed the greatest enrichment when using a buffer with an original pH of 9 (Table 2). Replacement of a carboxylic group (-COOH) with an aliphatic C (-CH_3_) implies a loss of 2 oxygen atoms and a gain of 2 hydrogen atoms, which gives a line with a slope of -1 in the van Krevelen diagram (red arrow in Fig. 2). Therefore, the line with a slope of about -1 in the van Krevelen diagram implies that carboxylic groups decrease from FA_pH3_ to FA_pH13_ (Fig. 2). The decrease in number of carboxylic groups as a function of elution sequence was also reported during extraction of the FA sub-fractions in the order of buffers, water, and ethanol in tandem^35^. Larger A_2920_/A_1720_ ratios with elution sequence suggested that LESF contained more aliphatic groups and less carboxylic/carbonyl groups (Table 1). The multi-peaks in the region of 1460–1360 cm^−1^ instead of peaks located at 1420 cm^−1^ indicated the prominence of aliphatic groups containing -C-H in LESF instead of carboxyl groups in EESF (Fig. 4). The lesser intensity of the peaks at 172 ppm showed that the EESF contained more carboxylic groups (Fig. 4). The decreasing content of carboxyl C was obtained by quantitative analysis of ^13^C-NMR (Table 2). When pH of the buffer was higher than pKa (8–10) of hydroxybenzenes, an increasing proportion of dissociated phenol is expected^5^,^18^. The largest content of phenolic C was observed in FA_pH9_ (Table 2). Using the buffer with the highest initial pH 13, the eluting sub-fractions showed weak polar components with methoxyl, O-alkyl, acetal and carbonyl groups instead of carboxyl and phenolic groups (Table 2). The various content of carboxylic and phenolic groups in FA sub-fractions is likely to affect the interactions with toxic metal ions.

No protein-like peaks were observed for standard FAs and HAs available from IHSS, or CSFA or even EESF in FTIR and fluorescence spectra (Figs. 2–3, Supplementary Fig. S2). However the appearance of amide bands in FTIR spectra and protein-like related peaks in fluorescence spectra, as well as the greater content of N indicated enrichment of protein-like components in LESF and FA_pH7_ (Figs. 2–3, Supplementary Fig. S2). The FTIR band typical of peptide structures in FA sub-fractions was also reported^5^. The appearance of peaks C and D, as well as the larger values of *P*_C_ and *P*_D_ further confirmed the existence of protein-like components in LESF. Peaks C and D have been widely observed for organic matter from lakes and seawaters^2^,^32^,^33^,^36^,^37^,^38^. Amino acids or protein-like components in river waters are bound with humic substances and this binding causes the high variability of the emission wavelength in fluorescence spectra^37^,^39^. Therefore, variability of the emission wavelength from FA_pH7_ to FA_pH13_ might be due to interactions between amino acids or protein-like components with humic substances (Table 3). Until now, this is the first report for possible enrichment of protein-like components with XAD-8 detected with fluorescence spectra. Protein-like components were recommended as a useful indicator of anthropogenic sources in river water^40^,^41^. However, Mostofa et al. documented the protein-like components probably came from both natural and anthropogenic sources in rivers^37^. Therefore, the origin and biogeochemical behaviors of protein-like components could to be further studied by isolation with the stepwise extractions.

The MM of CSFA sub-fractions decreased along the elution sequence according to size exclusion chromatography (Table 3). In fluorescence spectra, peaks A and B and were attributed to fulvic-like components with smaller MM and humic-like components with greater MM, respectively^2^,^32^,^33^,^42^,^43^,^44^. The strong decrease of *P_B_* with elution sequence was consistent with the decrease in average MM of CSFA sub-fractions with the elution sequence (Supplementary Fig. S3). The larger molecular mass dispersion for the LESF implied the more heterogeneous of humic substances^29^,^30^. The more heterogeneous structural compositions for LESF were further confirmed by the appearance of peaks C and D (Supplementary Fig. S2).

The amorphous and crystalline poly(methylene) groups have been recognized and reported in HA and humin with ^13^C-NMR spectra^45^,^46^,^47^. By using the stepwise elution method, the double-peaks corresponding to amorphous and crystalline poly(methylene) chains were observed at about 30 and 34 ppm in LESF (Fig. 4)^45^,^46^,^47^. To our knowledge, various amorphous and crystalline (CH_2_)_n_ chains have not been reported before in FA or its sub-fractions. The crystalline poly(methylene) regions are much less permissive than small molecules, however the mobile amorphous poly(methylene) regions can provide sorption sites for nonpolar contaminants^45^,^46^,^47^. However FAs are dissolved in larger pH range and no adsorption can be performed in natural water unless special procedure is employed. The possible influence of crystallites on the interactions or binding between FAs and contaminants should be investigated in the future. In addition, the crystallites were resistant to environmental attack and have long residence times^45^. Due to its exceptional biological stability, the crystalline component was recommended as an “internal standard” of the evolution of HAs^45^. The crystallites could also be used to research the possible evolution of FA, as another part of humic substance. This is a new perspective to the understanding of humic substances including FAs.

### Mechanism of stepwise elution

The EESF contained a greater proportion of carboxyl groups; however LESF had a greater proportion of phenolic groups evaluated with ^13^C-NMR spectra (Table 2). XAD-8 resin is basically hydrophobic although it contains slightly polar ester groups^21^. EESF which became sufficiently hydrophilic to be desorbed from the hydrophobic resin through ionization of carboxylic groups, were eluted by buffer with initial pH 3, 5, and 7. LESF which become sufficiently hydrophilic to be desorbed from the hydrophobic resin through ionization of phenolic groups, were eluted by buffer with initial pH 9 and 13^21^. That is, earlier elution with buffers of initial pH 3–7 was controlled by the deprotonation of carboxyl groups, while the later elution with buffer of initial pH 9–13 was dominated by deprotonation of phenolic groups. A similar phenomenon was reported with pH gradient desorption from XAD-8 resin^21^. In addition, simple model carboxylic and phenolic compounds were eluted at similar pH regions, which further confirmed those results^21^. The larger width of elution peaks for EESF could be attributed to the strong affinity for FA to XAD-8 at lower pH. The high mass proportion of EESF indicated that the ionization of carboxyl-related groups was likely one of the major features of CSFA that affected elution. The greater content of more easily dissociable sub-fractions of FAs were reported during sequential extractions of FAs from the Suwannee River and paddy soils^5^,^48^. As early as 1979, with pH gradient desorption coupled with IR spectra, MacCarthy et al. did not directly detect the presence or absence of phenolic groups in humic components eluted with buffer with pH 8–11^21^. Existences of higher content of phenolic groups in LESF, as a hypothesis was evaluated and confirmed with ^13^C-NMR spectra. Volumes of effluents before the elution peaks were less than those after elution peaks for all CSFA sub-fractions. Although not explicit in the literature, asymmetric peaks were observed in the figure during stepwise elution of FAs with various ratios of HCl and methanol^18^. Asymmetric peaks were also reported when FA sub-fractions were eluted in a stepwise process by use of universal buffers^5^. This should be further studied in the future.

Selection of proper standards to characterize the MM of humic substances is in large part determined by their hypothesized structure. With a mobile phase composition with an ionic strength equivalent to 1.0 M NaCl and a pH of 6.8, ploystyrene sulfonates can show a coiled configuration similar to humic substances, and be used as a reference material to examine the MM of humic substances^1^,^4^,^29^,^30^. This method has been successfully used to humic substances and naturally dissolved organic matter^1^,^4^,^29^,^30^. EESF had larger MM than LESF (Table 3). According to the theory of size exclusion, molecules of smaller nominal size can penetrate both small and large pores and thus would be elute later. However, larger molecules cannot access small pores and would be elute earlier^30^. Pore diameters of of size exclusion columns for chromatography commonly range from a few to several decades of nanometers, such as the YMC-Pack Diol-NP column, which has nominal pore sizes ranging from 6 to 30 nm (YMC Co., Ltd., Japan). The XAD-8 resin has an average pore diameter about 25 nm, which is approximately the pore diameter of size exclusion columns for chromatography columns. Therefore, the MM of CSFA sub-fractions decreased with elution sequence due to size exclusion. The preferential adsorption and hysteretic desorption of humic substances with smaller MM fraction in the case of porous adsorbents, such as activated carbon confirmed the effect of size exclusion^49^,^50^.

Proteins or protein-like components occur in soil and water. Proteins or protein-like components are amphoteric molecules that carry net positive and negative charges at a pH values less than and greater than their isoelectric point, respectively. Of the 600 common proteins more than 70% have isoelectric points greater than pH 5^51^. Thus, proteins or protein-like components were hydrophobic at pH less than 5 that make them adsorb to XAD-8 via electrostatic attraction and hydrophobic effect. However, at pH greater than 5, proteins or protein-like components were hydrophilic by ionization that resulted in them desorbing from XAD-8 via electrostatic repulsion. Therefore, the amino acids and/or proteins could be eluted and concentrated by the stepwise eluting procedure at pH7, 9, and 13.

In addition, the ash content in CSFA and its sub-fractions was at the same level with that in standard FAs from the Suwannee River, and was less than that in the standard FAs from soil or peat^52^. As an inorganic compound, the P content was less than ash content, which might indicate the negative effect on the composition or properties of CSFA and its sub-fractions (Table 1). Therefore, this technique could be applied to a wide variety of FAs from soils, sediments, and natural waters.

## Methods

### FA isolation

Surface soil (0–15 cm) was collected from the Jiufeng Mountain forest, Beijing, China. Soils were air-dried, ground to pass through a 2 mm mesh, and stored at 15°C before analyses. Isolation and purification of CSFA were performed by use of the XAD-8 resin method that has been recommended by IHSS^2^. Detailed information on the method has been previously reported^19^.

### Extraction and fractionation of CSFA sub-fractions

The 200 g CSFA was re-dissolved in pure water with soil-solution 1:10 (w/v), and then re-loaded to XAD-8 resin after adjusting to pH 1.0. Stepwise eluents were collected with buffers of pH 3, 5, 7, 9, and 13 by addition of volumes of NaOH and/or HCl to a 0.1 mol/L solution of sodium pyrophosphate. Eluents were collected per 50 mL. UV-vis absorbance at 650 nm was used to quantify CSFA and its sub-fractions during stepwise elution. After elution with each buffer, the eluate was adjusted immediately to pH 1.0 with 6 mol/L HCl. The acidified eluate was re-loaded onto the XAD-8 resin column and rinsed with distilled H_2_O (0.65 column volumes), and then with 0.1 mol/L NaOH (3 column volume). The eluate removed by use of NaOH was loaded onto an H^+^-saturated cation exchanged resin (Bio-Rad, Richmond, CA). Finally, purified CSFA and its sub-fractions were freeze-dried for chemical and spectroscopic analysis. Triplicate fractionations were performed with the above eluting procedure, and the results were reported as their average.

### Characterization of CSFA sub-fractions

Elemental composition (C, H, N, and S) was determined with an elemental analyzer (Elementar vario, macro EL, Germany) after vacuum drying at 60°C for 24 h. Ash content of CSFA and its sub-fractions was determined gravimetrically by loss of mass before and after 750°C for 6 h. Oxygen content was calculated by gravimetric difference. FTIR spectra of the CSFA and its sub-fractions were collected in the wavelength range of 400–4000 cm^−1^ using the KBr pellet method (Nicolet, US). Approximately 1.0 mg of each freeze-dried sample was mixed with potassium bromide (400 mg KBr) to prepare pellets for FTIR measurements. Solid-state ^13^C-NMR spectra were obtained with a Bruker Avance AV-400 (Bruker Corp., Billerica, MA) spectrometer at 100.62 MHz, with a repetition time of 110.7 ms, and an echo time of 4.5 ms.

Size exclusion chromatography was accomplished by use of an Aglient 1200 instrument with a YMC-pack Diol-60 column (YMC, Japan). The mobile phase of pH 6.8 comprised of 0.2 mol/L phosphate buffer and 1.0 mol/L NaCl was delivered at a flow rate of 0.5 mL/min^1^,^29^,^30^. The mobile phase was prepared with Milli-Q water and filtered through filter membranes with pore size 0.45 μm (Whatman, UK). The size exclusion chromatography column was calibrated by use of sodium polystyrene sulfonates as a reference material. The polystyrene sulfonates with MM of 10 kDa, 6.8 kDa, 4.3 kDa, and 210 Da, were purchased from the Sigma Company (Sigma, USA). The relative coefficient was 0.976 between the retention time and the logarithm of MM.

Fluorescence and UV-vis spectra were collected by use of scanning CSFA and its sub-fractions with concentration of 10 mg/L and 0.1 mol/L NaCl as medium. The control solution of 0.1 mol/L NaCl was prepared from Milli-Q water containing dissolved organic C < 0.1 mg/L. The fluorescence spectra were determined using a spectrofluorometer (Model F-7500; Hitachi, Japan) with a 150-W xenon lamp, a photomultiplier voltage 700 V, and a shutter control. Fluorescence spectra were recorded with 1200 nm/min and auto-response. Scan range were 200 to 450 nm for excitation (slit width 5 nm) and 200 to 600 nm for emission (slit width 10 nm). An inner-filter effect might interfere with correct interpretation of fluorescence peaks and percent fluorescence response because it reduces intrinsic fluorescence intensity^32^,^33^. An inner-filter correction was applied with the equations reported by Gauthier et al.^14^ and Pan et al.^53^. UV-Vis spectra were collected with Agilent 8453 (Agilent, US) with 200–800 nm.

## Supplementary Material

Supplementary Information Isolation and Characterization of Chinese Standard Fulvic Acid Sub-fractions Separated from Forest Soil by Stepwise Elution with Pyrophosphate Buffer

## Figures and Tables

**Table 1:** Elemental compositions, atomic ratios, double bond equivalent parameter, polarity ratio, and A_2950_/A_1720_ ratios of CSFA and its sub-fractions

	Mass percentages (%)							Atomic ratio			
Samples	C	H	O	N	S	Ash	TP	H/C	O/C	Polarity ratio (%)	A_2920_/A_1720_
FA_pH3_	44.23	3.81	47.79	3.11	0.59	0.49	0.26	1.03	0.81	87.1	0.51
FA_pH5_	46.96	4.48	44.72	2.96	0.63	0.23	0.26	1.14	0.71	76.9	0.52
FA_pH7_	49.52	5.40	40.83	3.29	0.61	0.32	0.06	1.30	0.62	67.6	0.52
FA_pH9_	52.60	6.10	36.32	4.18	0.53	0.28	0.17	1.38	0.52	58.6	0.59
FA_pH13_	53.04	6.19	36.04	4.20	0.54	0.34	0.03	1.39	0.50	57.4	0.63
CSFA	47.17	4.66	43.84	3.33	0.61	0.48	0.06	1.18	0.70	75.8	0.52

CSFA, Chinese standard fulvic acid from a forest soil; H/C, atomic ratio of hydrogen to carbon; O/C, atomic ratio of oxygen to carbon; Polarity ratio, atomic ratio of sum of N and O to C; TP, total phosphorous was measured with molybdenum blue method after digesting at 110°C and calculated as pyrophosphate.

**Table 2:** Distribution of carbon in CSFA and its sub-fractions calculated by solid-state ^13^C NMR spectroscopy

	Distribution of Carbon chemical shift (ppm)(%)								
	0–45	45–65	65–90	90–110	110–145	145–160	160–190	190–220	
Samples	Alkyl C	Methoxyl C	O-alkyl C	Acetal C	Aromatic C	Phenolic C	Carboxylic C	Carbonyl C	Aliphatic C ratio (%)
FA_pH3_	17.7	10.0	11.7	5.2	22.7	5.8	23.3	3.5	44.6
FA_pH5_	19.9	11.2	11.5	5.0	22	5.9	20.8	3.7	47.6
FA_pH7_	27.9	10.6	10.6	3.5	19.7	6.5	18.2	2.9	52.6
FA_pH9_	32.2	8.2	10.2	3.6	20.7	8.2	14.1	2.6	54.2
FA_pH13_	27.0	11.5	11.7	4.1	22.7	7.1	13.0	2.8	54.3
CSFA	22.7	10.1	10.1	3.9	21.4	5.5	22.0	4.2	46.8

Aliphatic C ratio: total aliphatic carbon region (0–110 ppm).

**Table 3:** Molecular mass and location of fluorescence peaks for CSFA and its sub-fractions

Samples	Number-averaged MM (Dalton)	Mass-averaged MM (Dalton)	Molecular mass dispersion	Peak A (230/410–415)^a^	Peak B (300–310)/(430–435)^a^	Peak C (270–275)/(305–340)^a^	Peak D (210–225)/(305–340)^a^
FA_pH3_	3814 ± 21	4432 ± 10	1.16	1200^b^	866^b^	-	-
FA_pH5_	3615 ± 27	4344 ± 26	1.20	910^b^	572^b^	-	-
FA_pH7_	3095 ± 91	3918 ± 89	1.27	898^b^	609^b^	185^b^	235^b^
FA_pH9_	1993 ± 81	3392 ± 73	1.70	541^b^	-	797^b^	639^b^
FA_pH13_	1754 ± 28	3369 ± 90	1.92	642^b^	-	420^b^	329^b^
CSFA	3625 ± 37	4338 ± 36	1.20	972^b^	720^b^	-	-

Molecular mass dispersion: ratio of mass-averaged MM to number-averaged MM.

^a^the location of fluorescence peak (excitation/emission nm/nm).

^b^fluorescence intensity for peaks per organic carbon (arbitrary unit); -: no peaks.
