# Supplementary material for: Isolation and Characterization of Chinese Standard Fulvic Acid Sub-fractions Separated from Forest Soil by Stepwise Elution with Pyrophosphate Buffer
Source: Sci Rep. 2015 Mar 4;5:8723. doi: 10.1038/srep08723 (PMC4348658; doi:10.1038/srep08723)
Supplement: Supplementary Information — Isolation and Characterization of Chinese Standard Fulvic Acid Sub-fractions Separated from Forest Soil by Stepwise Elution with Pyrophosphate Buffer [file srep08723-s1.docx]

Submitted to *Scientific Reports*

**Isolation and Characterization of Chinese Standard Fulvic Acid Sub-fractions Separated from Forest Soil by Stepwise Elution with Pyrophosphate Buffer**

Yingchen Bai^1^, Fengchang Wu^1^*, Baoshan Xing^2^, Wei Meng^1^, Guolan Shi^3^, Yan Ma^4^ & John P. Giesy^5,6,7,8^

^1^State Key Laboratory of Environmental Criteria and Risk Assessment, Chinese Research Academy of Environmental Sciences, Beijing, 100012, China; ^2^Stockbridge School of Agriculture, University of Massachusetts, Amherst, MA, 01003, USA; ^3^Lanzhou New Area Environmental Protection Bureau, Lanzhou, 730314, China; ^4^Research Center of Environmental Biology and Green Chemistry, School of Environmental and Municipal Engineering, Qingdao Technological University, Qingdao, 266033, China; ^5^Department of Biomedical and Veterinary Biosciences and Toxicology Centre, University of Saskatchewan, Saskatoon, Saskatchewan, Canada; ^6^Department of Biology and Chemistry, and State Key Laboratory for Marine Pollution, City University of Hong Kong, Kowloon, Hong Kong, China; ^7^State Key Laboratory of Pollution Control and Resource Reuse, School of the Environment, Nanjing University, Nanjing, 210046, China; ^8^Zoology Department, National Food Safety and Toxicology Center, and Center for Integrative Toxicology, Michigan State University, East Lansing, 48824, USA.

Correspondence and requests for materials should be addressed to:

F.C. ([wufengchang@vip.skleg.cn](mailto:wufengchang@vip.skleg.cn))

Number of pages: 5

Figure S1.


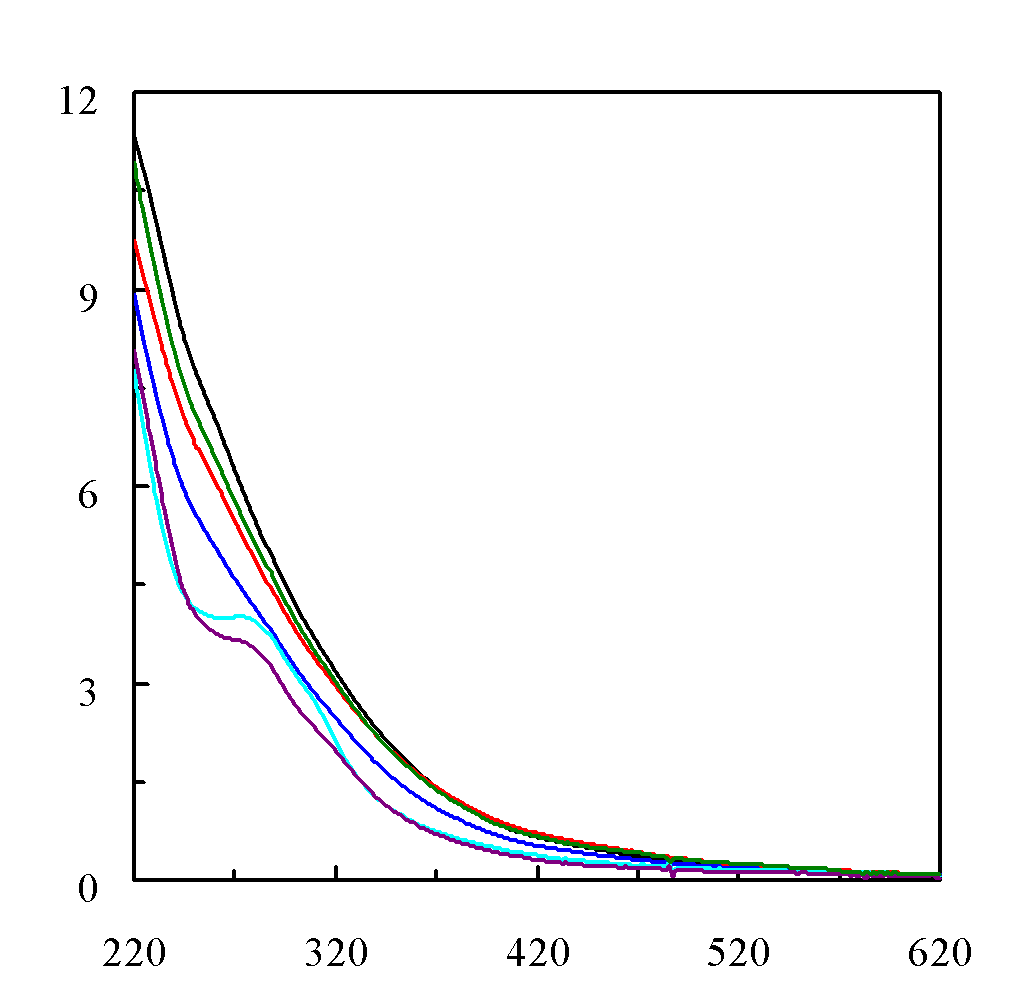



Figure S1. UV-Vis spectra of CSFA and its sub-fractions (10 mg/L).

Figure S2.


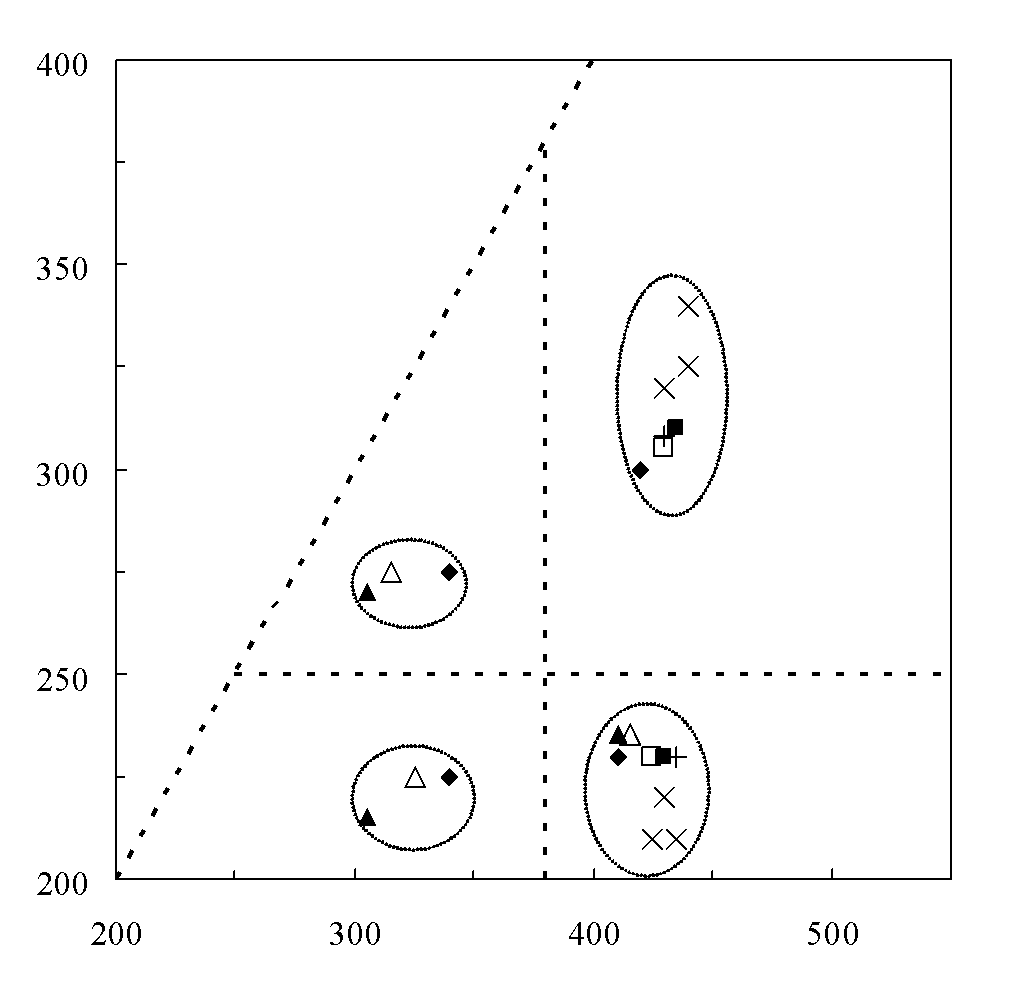


Figure S2. Location of fluorescence peaks of CSFA and its sub-fractions, as well as standard FAs of IHSS and operationally defined excitation and emission wavelength boundaries (dashed lines) for four regions. ×, standard FAs from IHSS; ■, FA_pH3_; □, FA_pH5_; ▲, FA_pH7_; Δ, FA_pH9_; and ♦, FA_pH13_. Standard FAs from IHSS includes Suwannee River I standard FA, Suwannee River II standard FA, Elliott Soil I standard FA, Elliott Soil II standard FA, Elliott Soil III standard FA, Pahokee Peat I standard FA, and Pahokee Peat II standard FA.

Figure S3.


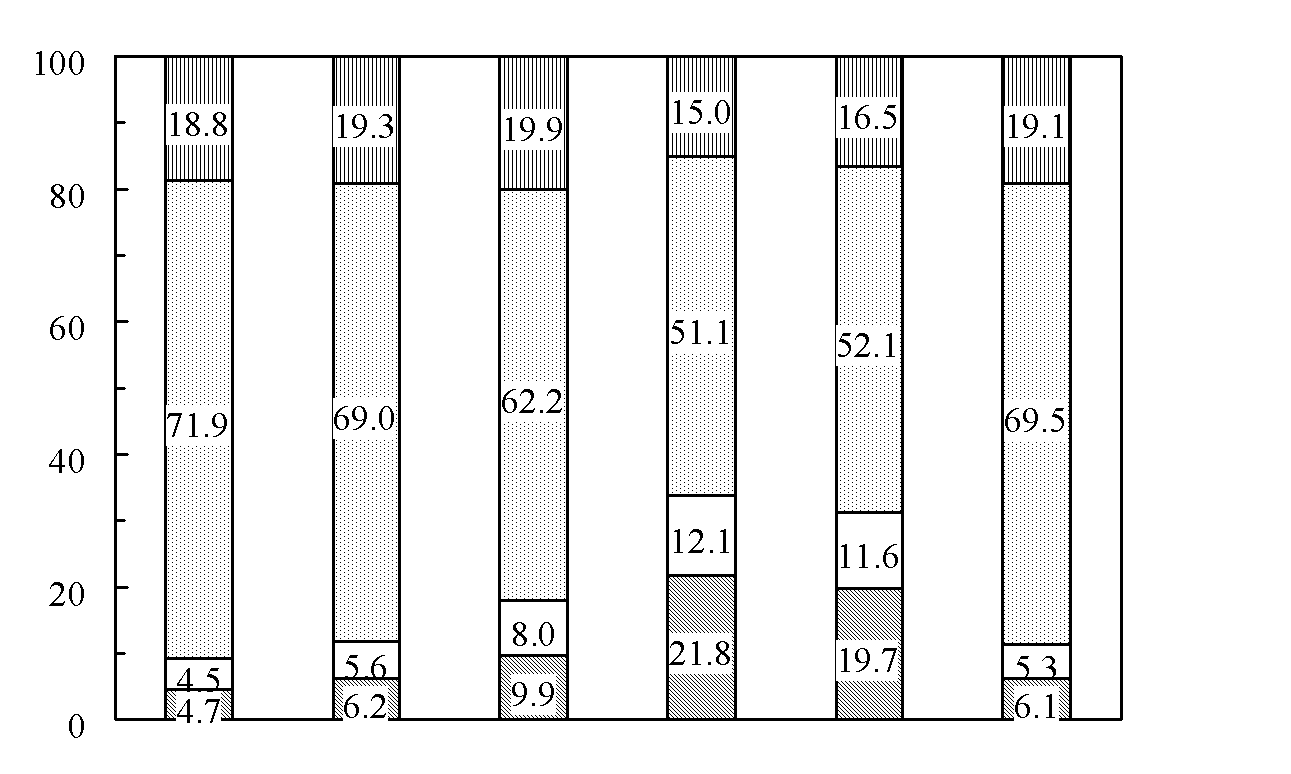


Figure S3. Percentage fluorescence response of CSFA and its sub-fractions.

- Peak A Region,
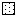
Peak B Region,
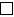
Peak C Region,
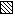
Peak D Region.

Table S1. Mass percentages of CSFA sub-fractions and parameters of elution curve from XAD-8 resin with buffers.

| Samples | Peak height | Peak width (L) | Mass percentages (%) |
| --- | --- | --- | --- |
| FA_pH3_ | 0.18 | 5 | 42.2±2.1 |
| FA_pH5_ | 1.06 | 3.7 | 36.3±1.7 |
| FA_pH7_ | 0.49 | 1.85 | 14.1±0.6 |
| FA_pH9_ | 0.22 | 1.55 | 5.0±0.3 |
| FA_pH13_ | 0.62 | 1.5 | 2.5±0.2 |

FA_pH3_, FA_pH5_, FA_pH7_, FA_pH9_, and FA_pH13_ are the sub-fractions eluted from the bulk CSFA using pyrophosphate buffer with initial pH 3, 5, 7, 9, and 13, respectively. The eluents were collected and detected the absorbance at 650 nm per 50 mL. Mass percentages were established by mass after freeze-drying.
